# Deep learning approach with ConvNeXt-SE-attn model for in vitro oral squamous cell carcinoma and chemotherapy analysis

**DOI:** 10.1016/j.mex.2025.103519

**Published:** 2025-07-17

**Authors:** Abhay Nath, Om Roy, Priyanka Silveri, Sanskruti Patel

**Affiliations:** a Department of Information Technology, Devang Patel Institute of Advance Technology and Research, Charotar University of Science and Technology, CHARUSAT Campus, Anand 388421, Gujarat, India; b Department of Information Technology, Smt. Kundanben Dinsha Patel Department of Information Technology, Charotar University of Science and Technology, CHARUSAT Campus, Anand 388421, Gujarat, India; c Department of Electrical and Computer Engineering, College of Engineering Drexel University, Philadelphia, PA, USA; d Smt. Chandaben Mohanbhai Patel Institute of Computer Applications, Charotar University of Science and Technology (CHARUSAT), Changa, 388421, Gujarat, India

**Keywords:** Oral Squamous Cell Carcinoma (OSCC), ConvNeXt, Grad-CAM, Squeeze-and-Excitation Blocks, Attention mechanisms, Deep learning

## Abstract

Oral squamous cell carcinoma (OSCC) continues to present a major worldwide healthcare problem because patients have poor survival outcomes alongside frequent disease returns. Globocan predicts that, OSCC will result in 389,846 new cases and 188,438 deaths globally during 2022 while maintaining an extremely poor 5-year survival rate at about 50%. Our method applies residual connections with Squeeze-and-Excitation blocks along with hybrid attention systems and enhanced activation functions and optimization algorithms to boost gradient movement throughout feature extraction. Compared against established conventional CNN backbones (VGG16, ResNet50, DenseNet121, and more), the proposed ConvNeXt-SE-Attn model outperformed them in all aspects of discrimination and calibration, including precision 97.88% (vs. ≤94.2%), sensitivity 96.82% (vs. ≤92.5%), specificity 95.94% (vs. ≤93.1%), F1 score 97.31% (vs. ≤93.8%), AUC 0.9644 (vs. ≤0.945), and MCC 0.9397 (vs. ≤0.910). The findings are critical to the increased feature-representation power and the robustness of classification of the architecture.

The proposed architecture employs ConvNeXt backbone with SE blocks and hybrid attention to extract essential details within class boundaries which standard models usually miss.

The activation through Gaussian-based GReLU incorporates Swish activation together with DropPath regularization for producing smooth gradient patterns which lead to generalizable features across imbalanced datasets.

Grad-CAM enhances interpretability by showing which image sections lead to predictions in order to enable clinical decisions.

The model demonstrates its capability as an effective detection method for minimal variations in oral cells which supports precise non-invasive treatment approaches for OSCC.

## Graphical abstract

**Figure:**
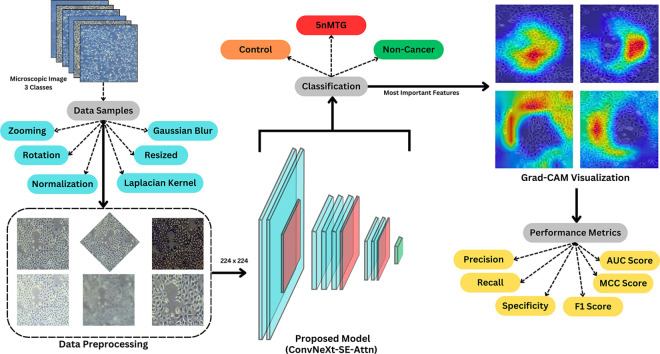

Specifications table

**Table:** 

Subject area	Computer Science and Applications
More specific subject area	Computer Vision
Name of your method	ConvNeXt-SE-Attn Model for In Vitro Oral Squamous Cell Carcinoma and Chemotherapy Analysis
Name and reference of original method	T. Gulsoy, E. Baykal Kablan, FocalNeXt: A ConvNeXt augmented FocalNet architecture for lung cancer classification from CT-scan images, Expert Syst. Appl. 261 (2025) 125,553.
Resource availability	Dataset: https://www.kaggle.com/datasets/abhaynath001/dataset

## Background

**Fig. 1:**
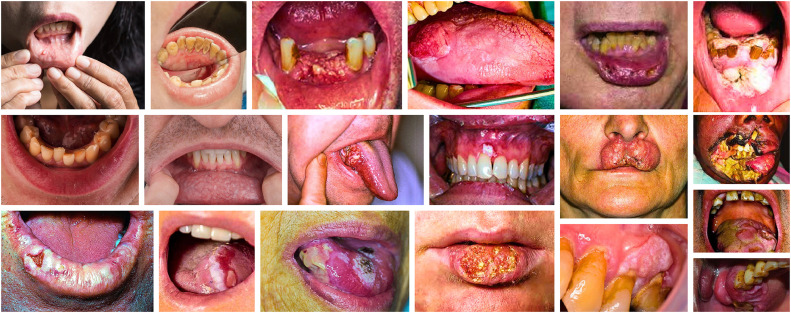
The clinical manifestations of oral cancer include ulcerative lesions, leukoplakia, erythroplakia, and invasive squamous cell carcinoma [[7], [8], [9]].

The global challenge of oral cancer, mostly targets oral squamous cell carcinoma (OSCC) as shown in Fig. 1, with a 5-year survival rate, because standard treatment options including surgery, radiotherapy, and chemotherapy tend to show limited effectiveness [1]. Globocan projects 389,846 new OSCC cases and 188,438 worldwide deaths for year 2022, highlighting oral squamous cell carcinoma (OSCC) as a significant contributor to global cancer incidence and mortality [2]. OSCC represents more than 90 % of oral malignancies, while showing an approximate 5-year survival rate of ∼50 %, which has demonstrated no significant reduction through therapeutically advanced medical systems [1,3]. The extensive use of tobacco, betel quid, and alcohol as risk factors, results in OSCC becoming a major socioeconomic burden that causes up to 25 % of all cancers in South Asian high-incidence areas [4]. The disease consequences of OSCC hit Asia particularly hard during 2020, since it accounted for 258,440 cases which represented two-thirds of total global OSCC incidents [2].

The diagnosis between untreated cells, resistant variants, and normal oral keratinocytes requires differentiation before personalized treatment strategies with effective solutions can be developed. The subtle morphological differences between these cell forms remain undetectable for traditional diagnostic methods such as manual histopathology and basic imaging, which results in delayed treatment or wrong diagnosis [4]. Vital staining using toluidine blue and autofluorescence imaging, aids in detecting suspect lesions but early-stage and chemoresistant cells tend to escape such subjective interpretation methods. The Global Cancer Observatory predicts that OSCC rates will expand by 40 % during the following two decades, thus compelling an urgent need for new detection instruments [2].

Chemoresistant cancer cell development constitutes a vital treatment challenge for OSCC, because these cells diminish standard therapeutic approaches and lead to unfavorable patient responses [3]. Precise diagnosis together with optimized patient treatment requires accurate population identification methods that separate untreated oral cancer cells from chemoresistant oral cancer cells and normal oral keratinocytes [5]. Convolutional neural networks (CNNs) represent a deep learning system which transformed medical imaging because they perform automated and highly accurate pattern recognition tasks [6].

The classification of cancer, uses deep learning algorithms such as VGG, AlexNet, DenseNet, ResNet and LeNet-5 according to previous research findings [[10], [11], [12], [13], [14]]. These models exhibit capability in discerning cancer from non-cancerous cells but experience difficulties differentiating chemoresistant from non-resistant cells within a single cell type. These networks hold architectural constraints which prevent them from capturing fine details within classes, therefore producing poor results when recognizing similar cell groups. These models experience various operational problems including gradient vanishing along with an inability to extract enough features and the risk of overfitting [[15], [16]]. The restricted usefulness of these methods prevents their adoption in actual clinical practice since healthcare teams need accurate resistance cell detection in order to optimize chemotherapy treatments.

Healthcare research regarding cancer has substantially progressed because deep learning models especially convolutional neural networks (CNNs) produce exact pattern recognition capabilities [6]. A better model design is necessary to handle classification difficulties, since it requires comprehensive multi-layer feature extraction capabilities. Current imaging analysis challenges faced by VGG16, AlexNet, DenseNet, ResNet, and LeNet-5 models can be solved through advanced modeling which allows better identification of patterns relevant to distinct micro-anatomical biological cell variations [17]. This advanced model design would deliver better outcomes than conventional architectural designs specifically when utilized in healthcare applications.

Our objective is to contribute new research utilizing deep learning for cancer diagnostics by conducting this study. The ConvNeXt-SE-Attn model represented our solution to classification problems by using advanced components which included residual connections [18], Squeeze-and-Excitation (SE) blocks [19], and hybrid attention mechanisms [20] for conducting feature extraction in multiple layers. The model benefits from our implementation of innovative activation functions alongside optimization approaches for optimizing gradient processing and performance levels. A precise classifier was combined with the usage of Grad-CAM [21] for visualizing regions which contribute to predictions within the cell lines. These progressions aim to show clinicians the different aspects of tumor diversity for more specific OSCC treatment plans which require non-physical methods.

The main contributions of this paper are summarized as follows:

• Integrated ConvNeXt-SE-Attn Architecture: The system integrates ConvNeXt backbone with Squeeze-and-Excitation (SE) blocks along with hybrid attention mechanisms to detect intra-class details which VGG and ResNet models typically overlook.
• Advanced Activation Functions: GReLU enables the model to incorporate Gaussian-based non-linearity functions with Swish activation which creates smoother gradients to help it identify complex and non-linear cell patterns and distinguish similar types of cells.
• Robust Regularization with DropPath: The method incorporates DropPath regularization where it randomly removes complete network segments for generating more generalized features that lower overfitting issues in highly unbalanced datasets.
• Enhanced Interpretability via Grad-CAM: The model uses Grad-CAM to visualize important areas in images which drive its prediction outcomes while enabling clinicians to make better decisions.

### Method details

The aim to develop the proposed method is to distinguish different cell populations, including treatment-sensitive and resistant phenotypes, using in vitro imaging data. We developed the ConvNeXt-SE-Attn model for the purpose of improving both feature extraction and classification accuracy. All steps from data preparation through preprocessing to model design, training, prediction, and heatmap visualization form the complete process. The process consists of stages that lead to effective classification together with visual decision-making explanations for interpretability purposes.

## Data collection

**Fig. 2:**
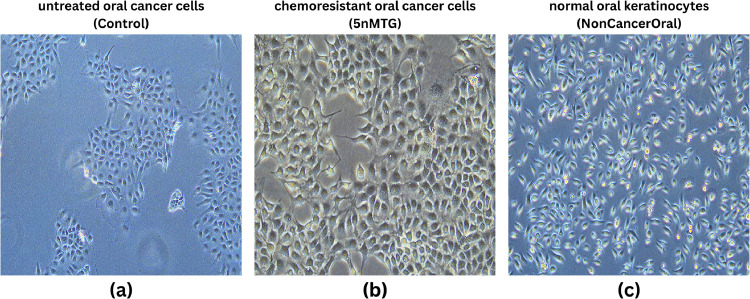
Sample images of the dataset used in this study (a) untreated oral cancer cells, (b) chemoresistant oral cancer cells, (c) normal oral keratinocytes.

We developed our own dataset that included three distinct types of oral cells which included untreated oral cancer cells, chemoresistant oral cancer cells (5nMTG), and normal oral keratinocytes (Fig. 2). The dataset contains three separate classes with 300 entirely distinct biological sample images for each class distribution. The untreated cancer cells serve as a baseline, while the chemoresistant variant identifies cells that can withstand 5nMTG treatment. Normal keratinocytes serve as a healthy control comparison. The in vitro data samples were collected from Indian Statistical Institute (ISI) Kolkata, using a 20X objective lens, with imaging captured at an appropriate wavelength (461 nm) for optimal cellular visualization. This curated dataset serves as the foundation for our model's training and evaluation, allowing us to distinguish subtle differences between these three important cell categories. This study makes available all in vitro microscopic images and dataset at https://doi.org/10.34740/kaggle/dsv/11633814 for model training and evaluation purposes.

## Data preprocessing

The dataset needed preprocessing to fulfill the input requirements of the deep learning model after image acquisition. Initial data partition included cancerous oral cells together with treatment-control cells and non-cancerous oral keratinocytes. The images received 224 × 224 pixels dimensions during resizing.

The dataset was split into two subsets:

• Training set (80 %): Used to learn patterns from the data.
• Validation set (20 %): Used to monitor model performance during training and detect overfitting.

Model generalization received improvement because all image pixel values underwent normalization by scaling into the [0, 1] range for stability during optimization. The mathematical expression of normalization appears as shown in Eq. 1:

(1) $$X_{\text{norm}}=\frac{X_{\text{raw}}}{255}$$

The normalization process transforms pixel values to X_norm_ while the raw pixel intensity X_raw_ spans from 0 to 255.

**Fig. 3:**
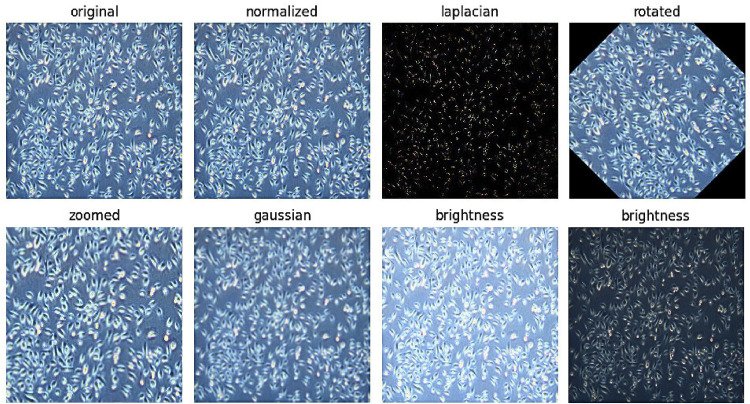
Different preprocessing techniques applied on the original dataset.

The dataset improvement process included multiple focused methods to optimize its quality. A Laplacian kernel function extracted image details and edges because it improves feature detection. Our protocol involved controlled adjustments of image rotation along with zoom parameter changes to emulate various camera viewing angles and camera distance positions. Gaussian blurring produced softening, that caused a blurry unsharpness effect on the images as brightness adjustments simulated numerous possible lighting situations. The model gained knowledge of consistent and broad patterns by incorporating these authentic transformations to images (Fig. 3).

## **Proposed** ConvNeXt-SE-Attn **model**

**Fig. 4:**
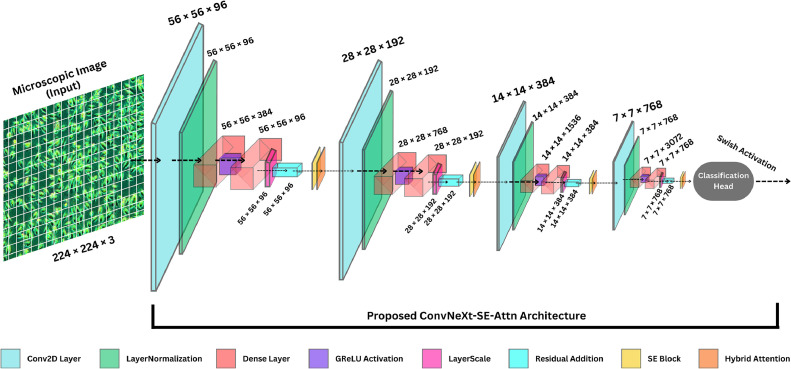
Architecture of proposed ConvNeXt-SE-Attn model.

**Fig. 5:**
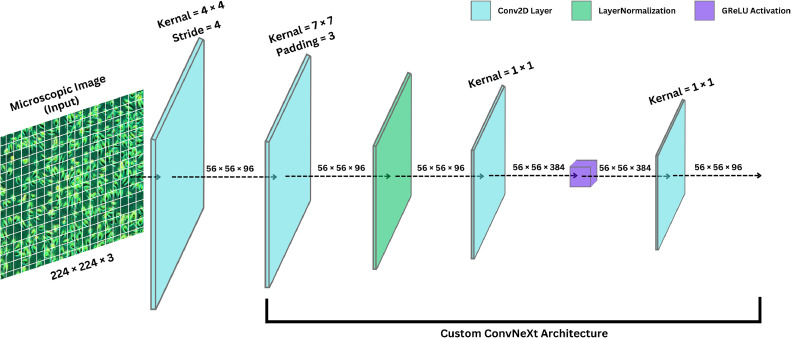
Architecture of custom ConvNeXt backbone model.

In this research, we developed a deep learning-based classification model, ConvNeXt-SE-Attn (Fig. 4), for identifying oral squamous cell carcinoma (OSCC) and related cell types. The first step involved using ConvNeXt (Fig. 5) as the backbone since it contains state-of-the-art pre-trained CNN features with hierarchical elements for progressive downsampling, large kernel convolutions. and residual connections alongside layer normalization for efficient multi-scale feature extraction [22]. A modified version of the ConvNeXt model received our customization improvements for OSCC classification while we integrated customized layers for the particular task. New architectural modifications were added to improve model generalization as well as decrease overfitting problems. We substituted traditional fully connected layers with global average pooling to summarize spatial features in the feature maps during dimension reduction [23].

A layer with 128 neurons followed for extracting sophisticated data patterns from the information. The GReLU (Gaussian ReLU) activation function served as an enhancement to the learning process in this layer. The GReLU activation function was employed because its Gaussian-based non-linearity helps achieve better gradient flow and feature extraction for complex oral cell images as expressed in Eq. 2 [24]:

(2) GReLU(x)=N(0,1)×max⁡(0,x)

Where N(0,1) denotes a Gaussian distribution. The non-linear activation enables the model to discover diverse features which strengthens its performance in understanding complex feature interconnections [25].

The model gained improved channel-wise feature recalibration and spatial context abilities through the integration of Squeeze-and-Excitation (SE) blocks with hybrid attention mechanisms after each stage. We applied Swish activation to the output layer before softmax activation because its non-monotonic smooth behavior enhances gradient flow thus enabling better detection of complex patterns according to Eq. 3 [26]:

(3) Swish(x)=x·σ(x)

The output activation function in this context is sigmoid function denoted as σ(x). Swish activation improves model performance by creating smooth gradients which enable easier learning, particularly in deep structures [27].

We utilized DropPath regularization to make the model more robust while fighting against overfitting during training. Training the model with DropPath allows the removal of complete network paths termed blocks through stochastic methodology as defined in Eq. 4 [28]. The method makes the model build stronger diverse representation patterns so it becomes less reliant on particular network elements and demonstrates enhanced generalization.

(4) $$\tilde{x}=\frac{x⊙b}{1-\lambda }$$

The equation represents the DropPath process which applies binary mask b ∼ Bernoulli, (1 − λ) to the entire network path with input tensor x while using drop rate λ.

Table 1 summarizes the proposed architecture for the ConvNeXt-SE-Attn model, a modified ConvNeXt-Small variant. It outlines key components including Swish activation, SE blocks, and attention mechanisms. The model comprises 49.55 M parameters and features four stages of convolutional blocks with sequential downsampling and a final dense classification head. Parameter details and layer configurations are provided to facilitate comprehension of the design choices.

**Table 1:** Model summary of proposed ConvNeXt-SE-Attn architecture.

Layer Type		Number of Filters	Size of Feature Map (Height × Width × Channel)	Size of Kernal	Number of Stride	Number of Padding
Image Input Layer			224 × 224 × 3			
Stage 0	1st convolution layer	96	56 × 56 × 96	4 × 4	4	
	LayerNormalization	96	56 × 56 × 96			
	Dense layer	384	56 × 56 × 384			
	GReLU	96	56 × 56 × 96			
	Dense layer	96	56 × 56 × 96			
	LayerScale	96	56 × 56 × 96			
	Residual Addition	96	56 × 56 × 96			
Stage 1	2nd convolution layer	192	28 × 28 × 192	3 × 3	2	1
	LayerNormalization	192	28 × 28 × 192			
	Dense layer	768	28 × 28 × 768			
	GReLU	192	28 × 28 × 192			
	Dense layer	192	28 × 28 × 192			
	LayerScale	192	28 × 28 × 192			
	Residual Addition	192	28 × 28 × 192			
Stage 2	3rd convolution layer	384	14 × 14 × 384	7 × 7	1	3
	LayerNormalization	384	14 × 14 × 384			
	Dense layer	1536	14 × 14 × 1536			
	GReLU	384	14 × 14 × 384			
	Dense layer	384	14 × 14 × 384			
	LayerScale	384	14 × 14 × 384			
	Residual Addition	384	14 × 14 × 384			
Stage 3	4th convolution layer	768	7 × 7 × 768	3 × 3	2	1
	LayerNormalization	768	7 × 7 × 768			
	Dense layer	3072	7 × 7 × 3072			
	GReLU	768	7 × 7 × 768			
	Dense layer	768	7 × 7 × 768			
	LayerScale	768	7 × 7 × 768			
	Residual Addition	768	7 × 7 × 768			
Classification Head						
GlobalAveragePooling2D			1 × 1 × 768			
Dense + Swish / GReLU + DropPath		128				
Dense (Softmax)		3				

## Model training

AdamW served as the optimizer during training because it integrates weight decay to enhance the regularization process. During gradient-based adjustment of learning rates the AdamW optimizer improved speed to convergence without causing overfitting [29]. There are three components in the parameter θ_t_ update rule at time step t as shown in Eq. 5:

$$m_{t}=\beta_{1}m_{t-1}+\left(1-\beta_{1}\right)g_{t}$$

$$v_{t}=\beta_{2}v_{t-1}+\left(1-\beta_{2}\right)g_{t}^{2}$$

$${\hat{m}}_{t}=\frac{m_{t}}{1-\beta_{1}^{t}},\;{\hat{v}}_{t}=\frac{v_{t}}{1-\beta_{2}^{t}}$$

(5) $$\theta_{t+1}=\theta_{t}-\eta \frac{{\hat{m}}_{t}}{\sqrt{{\hat{v}}_{t}}+\epsilon }+\lambda \theta_{t}$$

The update rules for θ_t_ parameter at time step t include first and second moment estimates m_t_ and v_t_ respectively, with learning rate η, weight decay factor λ, and small constant ϵ for preventing zero division.

The training procedure adopted categorical cross-entropy because it works best for multi-class classification [30]. The mathematical representation of the loss function appears in Eq. 6:

(6) $$L=-\sum_{i=1}^{n}y_{i}\log\left({\hat{y}}_{i}\right)$$

Where n is the number of classes, y_i_ is the true label, and ${\hat{y}}_{i}$ is the predicted probability for class i.

The training model was run for 350 epochs using the loss and accuracy values evaluated on training and validation data. The trained model received preservation for maintaining its optimal parameters that will support future prediction operations.

## Heatmap-based visualization using grad-cam

The predictions from our model received clarification through the use of Gradient-weighted Class Activation Mapping (Grad-CAM). Grad-CAM generates heatmaps which mark down specific image regions that the model uses for its classification determination [31]. The visual outputs from this step indicated whether the model analyzed beneficial biological cell image regions.

The process of Grad-CAM calculates the gradient of output class scores against the feature maps found in the final convolutional layer [32]. A single weight is obtained from pooling gradient values through this process. The features maps receive weighted summation to manufacture the heatmap using these weights. The Grad-CAM algorithm can be summarized as follows:

1. y_c_ (the output score for class c) must be calculated by taking the derivative of the feature map activations A^k^.
2. The weight computation for each feature map follows the pooling operation (Eq. 7):
  (7) $$\alpha_{k}^{c}=\frac{1}{z}\sum_{i}\sum_{j}\frac{\partial y^{c}}{\partial A_{\text{ij}}^{k}}$$

The number of pixels in each feature map is represented by variable Z.

3. The feature maps are summed through their weighted values according to the following computation (Eq. 8):
  (8) $$L_{\text{Grad}-\text{CAM}}^{c}=\text{ReLU}\left(\sum_{k}\alpha_{k}^{c}A^{k}\right)$$

The ReLU activation function allows positive values in the heatmap to be included.

The heatmap received dimension adjustment for achieving input image dimensions and later displayed on top of the original image through a transparent layer. The visualization tool revealed which areas of the image the model identified as crucial for its decision-making process. Grad-CAM proved to be a vital diagnostic instrument because it enabled clinical users to better understand the choices made by the model during operation.

### Model validation

The following are the validation details of the proposed ConvNeXt-SE-Attn model.

1. Experimental Setup

**Hardware Environment:** The AI model underwent rigorous evaluation on a NVIDIA DGX STATION A100 computer, leveraging its advanced specifications to maximize performance potential (Table 2) [33].

**Table 2:** NVIDIA DGX STATION A100 hardware environment specifications.

Specification	Details
Processor	Single AMD 7742, 64 Cores, operating at 2.25 GHz (Base) - 3.4 GHz (Max Boost)
System Memory	512 GB DDR4
GPU	4 NVIDIA A100 GPUs with 40 GB VRAM each
Performance	Capable of achieving 2.5 Peta FLOPS AI and 5 Peta OPS INT8
GPU Memory	160 GB
Total System Storage	1×1.92 TB NVME Drive
Internal Storage	7.68 TB U.2 NVME Drive
System Network	Dual-port 10Gbase-T Ethernet LAN and a Single-port 1 Gbase-T Ethernet BMC Management Port
Operating System	Ubuntu Linux
System Power Usage	Operates efficiently at 1.5 kW under 100–120 Vac
Display	Features 4GB GPU Memory and supports 4x Mini DisplayPort
Operating Temperature	Maintained within 5–35°C (41–95°F)

**Software Environment:** The development of the AI model used Python as its main programming language while TensorFlow acted as the base machine learning framework alongside Keras for designing neural networks at an advanced level. A wide range of packages within this software environment included data manipulation tools, Pandas, NumPy, and visualization tools like Matplotlib and Seaborn. The architecture combines VGG16, VGG19, ResNet50, ResNet152, InceptionResNetV2, GoogLeNet, DenseNet121, NASNet, EfficientNetB0, MobileNetV2, a custom CNN, and U-Net network designs with optimal customization for diverse domains.

**Hyperparameter Details and Training Parameters:** The proposed model used a ConvNeXtSmall backbone, pre-trained on ImageNet, taking 224 × 224 RGB images as input. The backbone is frozen to leverage existing learned features. A GlobalAveragePooling2D layer then reduces the spatial dimensions, feeding into a Dense layer with 128 units that compresses and integrates features. We use a custom GReLU activation to add Gaussian-based non-linearity, replacing the standard GeLU, and improve feature learning. A DropPath layer with a rate of 0.2 is applied to randomly drop paths during training, which helps reduce overfitting. The final Dense layer used a swish activation function in the classification head before softmax layer to classify images into three categories. For training, we use the AdamW optimizer with a learning rate of 3 × 10^−3^ for steady weight updates and a weight decay of 1 × 10^−4^ to regularize the model. A batch size of 32 is used, and data augmentation is applied to improve generalization.

**Fig. 6:**
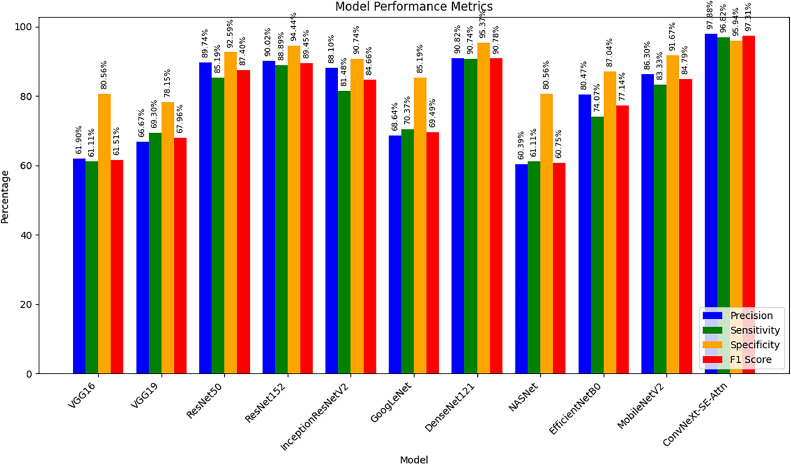
Different model comparisons between best epoch and learning rate.

Table 3 shows the training settings used for different deep learning models. For each model, it lists the learning rate, the activation function used in the hidden layer (mostly ReLU, except one with GReLU + Swish), the output activation function (Softmax), the optimizer, and the best epoch number where the model achieved its peak performance. For example, VGG16 and VGG19 use learning rates of 2×10⁻⁵ and 1×10⁻⁵ respectively, with ReLU activations in hidden layers and softmax for the output, optimized using Adam, reaching optimal performance at 52 and 78 epochs. ResNet50 and ResNet152 employ learning rates of 5×10⁻⁵ and 1×10⁻⁵ respectively, with best epochs at 14 and 188. InceptionResNetV2 follows a similar configuration with a 2×10⁻⁵ learning rate achieving peak performance at 69 epochs, whereas GoogLeNet and NASNet use lower learning rates (8×10⁻⁶ and 1×10⁻⁵) and converge at 206 and 192 epochs. EfficientNetB0 and MobileNetV2 use intermediate learning rates (4×10⁻⁵ and 7×10⁻⁵) and peak at 61 and 103 epochs. In contrast, the proposed ConvNeXt-SE-Attn model utilizes a considerably higher learning rate of 3×10⁻³, combines advanced activations (GReLU and Swish), employs the AdamW optimizer, and reaches its optimal performance at 312 epochs, reflecting its greater complexity and enhanced feature extraction capabilities (Fig. 6).

**Table 3:** Training parameters details of different models.

Model	Learning Rate	Activation (Hidden Layer)	Activation (Output Layer)	Optimizer	Best Epoch
VGG16	2 × 10^−5^	ReLU	Softmax	Adam	52
VGG19	1 × 10^−5^	ReLU	Softmax	Adam	78
ResNet50	5 × 10^−5^	ReLU	Softmax	Adam	14
ResNet152	1 × 10^−5^	ReLU	Softmax	Adam	188
InceptionResNetV2	2 × 10^−5^	ReLU	Softmax	Adam	69
GoogLeNet	8 × 10^−6^	ReLU	Softmax	Adam	206
DenseNet121	3 × 10^−5^	ReLU	Softmax	Adam	77
NASNet	1 × 10^−5^	ReLU	Softmax	Adam	192
EfficientNetB0	4 × 10^−5^	ReLU	Softmax	Adam	61
MobileNetV2	7 × 10^−5^	ReLU	Softmax	Adam	103
ConvNeXt-SE-Attn (Proposed)	3 × 10^−3^	GReLU + Swish	Softmax	AdamW	312

## Results and discussions

To evaluate the performance of the proposed ConvNeXt-SE-Attn model, benchmark performance evaluation metrics are used.

Precision quantifies the proportion of predicted positive instances that are truly relevant, thereby measuring the accuracy of positive forecasts [34]. It is defined in Eq. 9:

(9) $$\text{Precision}=\frac{\text{TP}}{\text{TP}+\text{FP}}$$

Recall (Sensitivity) actions the capability to correctly detect all relevant positive instances [34]. It is defined in Eq. 10:

(10) $$\text{Sensitivity}=\frac{\text{TP}}{\text{TP}+\text{FN}}$$

Specificity measures the ability to correctly identify negative instances, indicating how well the model avoids false positives [34]. It is defined in Eq. 11:

(11) $$\text{Specificity}=\frac{\text{TN}}{\text{TN}+\text{FP}}$$

The F1-score is the harmonic mean of recall and precision, balancing these two metrics [34]. It is defined in Eq. 12:

(12) $$F1\;\text{Score}=2\times \frac{\text{Precision}\times \text{Recall}}{\text{Precision}+\text{Recall}}$$

Mean Average Precision (mAP) measures the average precision across multiple queries or categories, assessing ranking performance [34]. It is defined in Eq. 13:

(13) $$\text{mAP}=\frac{1}{N}\sum_{i=1}^{N}AP_{i}$$

### Performance evaluation

Table 4 represents the performance of the proposed *ConvNeXt-SE-Attn model* with comparison to other benchmark models includes VGG [35], ResNet [36], GoogLeNet [37], DenseNet [38], NASNet [39] etc. The performance was significantly better than previous models after the proposed ConvNeXt-SE-Attn model architecture was developed and trained (Table 4).

**Table 4:** The performance evaluation of proposed ConvNeXt-SE-Attn model with different deep learning models.

Model	Precision	Sensitivity	Specificity	F1 Score
VGG16	61.90 %	61.11 %	80.56 %	61.51 %
VGG19	66.67 %	69.30 %	78.15 %	67.96 %
ResNet50	89.74 %	85.19 %	92.59 %	87.40 %
ResNet152	90.02 %	88.89 %	94.44 %	89.45 %
InceptionResNetV2	88.10 %	81.48 %	90.74 %	84.66 %
GoogLeNet	68.64 %	70.37 %	85.19 %	69.49 %
DenseNet121	90.82 %	90.74 %	95.37 %	90.78 %
NASNet	60.39 %	61.11 %	80.56 %	60.75 %
EfficientNetB0	80.47 %	74.07 %	87.04 %	77.14 %
MobileNetV2	86.30 %	83.33 %	91.67 %	84.79 %
ConvNeXt-SE-Attn (Proposed)	97.88 %	96.82 %	95.94 %	97.31 %

Table 4*compares the performance of various deep learning models. Standard architectures like VGG16, VGG19, and NASNet achieve moderate precision around 60–67 %, while advanced models such as ResNet50, ResNet152, and DenseNet121 perform better with precision values close to 90 %. However, the proposed ConvNeXt-SE-Attn model outperforms all others with a precision of 97.88 %, sensitivity of 96.82 %, specificity of 95.94 %, and an F1 score of 97.31 %. These outcomes demonstrate how well our customized architecture performs in terms of memory, general resilience, and precision while categorizing* in vitro *microscopic pictures. Despite the disparity in classes, these results are essential because reducing false positives minimizes unnecessary treatments, while lowering false negatives ensures no malignant cells are overlooked, thereby guaranteeing more accurate identification of cancerous cells.*

Table 5 *includes the 95 % confidence intervals (CIs) and p-values of all the performance metrics that allow one to compare each of the baseline models with the proposed ConvNeXt-SE-Attn model. Using the normal approximation of the binomial distributions proportions, the CIs define the bounds in which the actual population measure falls with a 95 % assurance of accuracy. To illustrate, the precision level of the proposed model (97.88 %) is equated to CI (96.94 - 98.82 %), thus signifying the low variability and statistical reliability of the research data. In comparison, VGG16 simulates considerably broader CI (58.73 - 65.07 %), indicating less reliable, and thus lower performance.*

**Table 5:** Performance comparison of proposed ConvNeXt-SE-Attn model with baseline models including 95 % confidence intervals (CIs) and p-values based on two-proportion z-tests.

Model	Precision (95 % CIs)	p-value	Sensitivity (95 % CIs)	p-value	Specificity (95 % CIs)	p-value	F1 Score (95 % CIs)	p-value
VGG16	58.7 – 65.1 %	9.37×10^−101^	57.9 – 64.3 %	5.61×10^−95^	78.0 – 83.1 %	1.74×10^−25^	58.3 – 64.7 %	2.07×10^−97^
VGG19	63.6 – 69.7 %	1.87×10^−80^	66.3 – 72.3 %	7.98×10^−63^	75.5 – 80.8 %	2.18×10^−31^	64.9 – 71.0 %	4.28×10^−71^
ResNet50	87.8 – 91.7 %	3.59×10^−13^	82.9 – 87.5 %	1.29×10^−18^	90.9 – 94.3 %	2.18×10^−3^	85.2 – 89.6 %	8.09×10^−16^
ResNet152	88.1 – 92.0 %	1.34×10^−12^	86.8 – 90.9 %	6.22×10^−15^	92.9 – 95.9 %	1.37×10^−1^	87.4 – 91.5 %	1.11×10^−11^
InceptionResNetV2	86.0 – 90.2 %	1.24×10^−16^	78.9 – 84.0 %	3.66×10^−11^	88.8 – 92.6 %	8.65×10^−06^	82.3 – 87.0 %	7.47×10^−22^
GoogLeNet	65.6 – 71.7 %	6.99×10^−73^	67.4 – 73.4 %	1.20×10^−73^	82.9 – 87.5 %	2.08×10^−15^	66.5 – 72.5 %	1.46×10^−65^
DenseNet121	88.9 – 92.7 %	5.25×10^−11^	88.8 – 92.6 %	5.53×10^−1^	94.0 – 96.7 %	5.53×10^−1^	88.9 – 92.7 %	3.42×10^−09^
NASNet	57.2 – 63.6 %	7.82×10^−108^	57.9 – 64.3 %	5.61×10^−95^	78.0 – 83.1 %	1.74×10^−25^	57.6 – 63.9 %	7.19×10^−101^
EfficientNetB0	77.9 – 83.1 %	3.24×10^−35^	71.2 – 76.9 %	7.19×10^−12^	84.8 – 89.2 %	7.19×10^−12^	74.4 – 79.9 %	3.25×10^−41^
MobileNetV2	84.1 – 88.5 %	1.18×10^−20^	80.9 – 85.8 %	1.62×10^−4^	89.9 – 93.5 %	1.62×10^−04^	82.4 – 87.1 %	1.49×10^−21^
ConvNeXt-SE-Attn (Proposed)	96.9 – 98.8 %	—	95.7 – 98.0 %	—	94.7 – 97.2 %	—	96.3 – 98.4 %	—

P-values were derived using two-sample z-tests (often referred to as a variant of the *t*-test under large sample sizes, here n = 900), assuming independent samples. These quantify the statistical significance of differences between the proposed model and each baseline. Very small p-values are a signal that the performance gains achieved are not likely to be due to chance; i.e., the p-value of VGG16 precision is 9.37×10^−101^ and NASNet precision p-value is 7.82×10^−108^. Together, the small CIs and the lower values of p with all the metrics, support the superiority of the proposed model in statistical evaluation over the traditional architectures, which makes it robust and in a much better position to classify.

### Activation function comparison

Table 6 *compares seven activation functions in the ConvNeXt-SE-Attn model. It lists the mean average precision (mAP), validation precision, and validation loss for each function. Swish performs the best, achieving the highest mAP (97.73 %) and validation precision (97.88 %) with very low loss (0.39 %). Mish, Leaky ReLU, ReLU, and GReLU show similar, strong performance, while Tanh is slightly lower. Sigmoid function has much lower mAP and precision, along with a high loss. The 95 % confidence intervals confirm the stability of Swish and other top activations, while p-values show that only Sigmoid differs significantly, validating Swish’s superiority.*

**Table 6:** Comparison of the precision of 7 activation functions in ConvNeXt-SE-Attn model with 95 % confidence intervals (CIs) and p-values based on two-proportion z-tests.

Activation Function	Mean Average Precision (mAP)	Validation Precision	Validation Precision (95 % CIs)	p-value	Validation Loss
Swish	97.73 %	97.88 %	96.94 – 98.82 %	—	0.39 %
Mish	97.30 %	97.78 %	96.83 – 98.73 %	0.7902	0.71 %
Leaky ReLU	97.19 %	97.75 %	96.79 – 98.71 %	0.7126	1.56 %
ReLU	97.14 %	97.71 %	96.75 – 98.67 %	0.5964	1.45 %
Tanh	96.62 %	97.57 %	96.58 – 98.56 %	0.2417	1.74 %
GReLU	97.36 %	97.72 %	96.76 – 98.68 %	0.6251	1.64 %
Sigmoid	46.77 %	71.68 %	68.75 – 74.61 %	6.83×10^−107^	8.43 %

### Classification and visualization with Grad-CAM

**Fig. 7:**
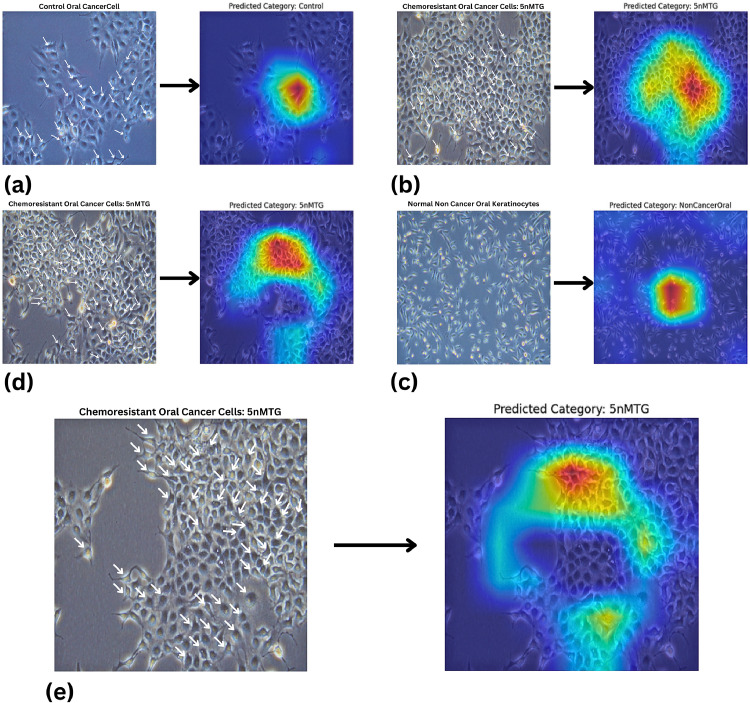
Visual results illustrating the model’s predictions and Grad-CAM heatmaps, highlighting key features in various oral cell images.

Fig. 7 illustrate how the model classifies different types of oral cells and how Grad-CAM highlights the critical features guiding each prediction. The heatmaps show the regions the model focuses on, with warmer colors (red and yellow) indicating higher importance, and cooler colors (blue) showing less influence. This helps us understand which parts of the image led the model to predict a specific class, whether it’s control cells, chemoresistant cells, or non-cancer cells. By visually confirming these important regions, we gain confidence in the model’s decision-making process and see how it identifies subtle differences among cell types.

In Fig. 9, sample (a) represents a control oral cancer cells, correctly classified as part of the control class. The Grad-CAM visualization highlights its most important features in warm colors (red and yellow), indicating the region’s most responsible for the classification. Samples (b), (d), and (e) are chemoresistant oral cancer cells, all accurately classified under the 5nMTG class, with their key distinguishing features similarly highlighted in warm tones. Sample (c) shows a non-cancerous normal oral cell, correctly classified as part of the non-cancer class. Across all samples, cooler colors (such as blue) indicate regions with lower importance in the classification decision, providing insight into how the model focuses on specific morphological features for accurate prediction.

### Comprehensive model evaluation with visuals

**Fig. 8:**
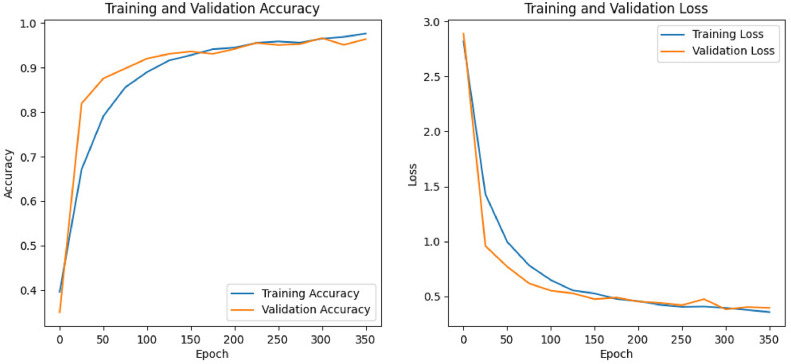
Training and validation accuracy (left) and loss (right) over epochs, demonstrating strong convergence with minimal overfitting.

The left graph shows training and validation accuracy rising rapidly, then leveling off near 98 %. The right graph shows both losses dropping sharply early on, then gradually flattening at low levels (Fig. 8). The close alignment of the training and validation curves indicates minimal overfitting and strong overall performance.

**Fig. 9:**
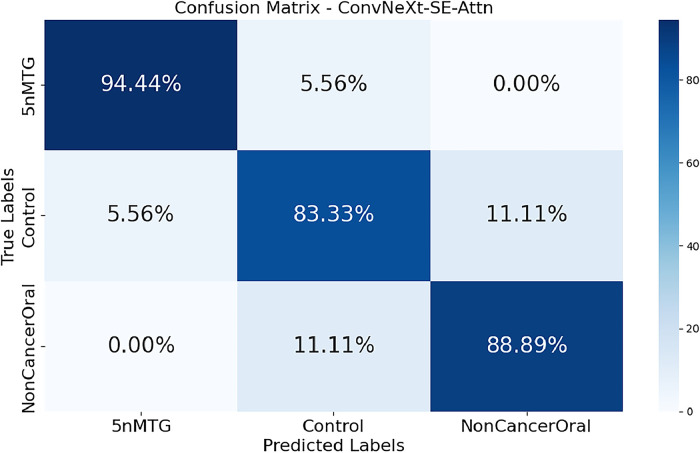
Confusion matrix of the ConvNeXt-SE-Attn model, illustrating classification performance across different oral cell categories.

The confusion matrix (Fig. 9) provides insight into the model's classification performance across the three cell types. The high diagonal values indicate strong accuracy in correctly identifying each class, with minimal misclassification. The ConvNeXt-SE-Attn model demonstrates particularly strong differentiation between normal oral cells, untreated oral cancer cells, and chemoresistant oral cancer cells (5nMTG). Misclassifications were minimal and comprised not more than 11.11 % of the total samples. In particular, 5.56 % of the chemoresistant (5nMTG) cells were wrongly identified as the control cells. Among the control class, 5.56 % and 11.11 % were falsely classified as chemoresistant (5nMTG) and non-cancerous, respectively. In the non-cancerous category, 11.11 % against the control were in error. The errors seem to be brought about by low morphological resemblance between the cell types, especially the control cells and non-cancerous cells, whose morphology may share common features to illustrate an overlap when viewed under the microscope. Despite this, the model demonstrates strong overall accuracy and class separation.

**Fig. 10:**
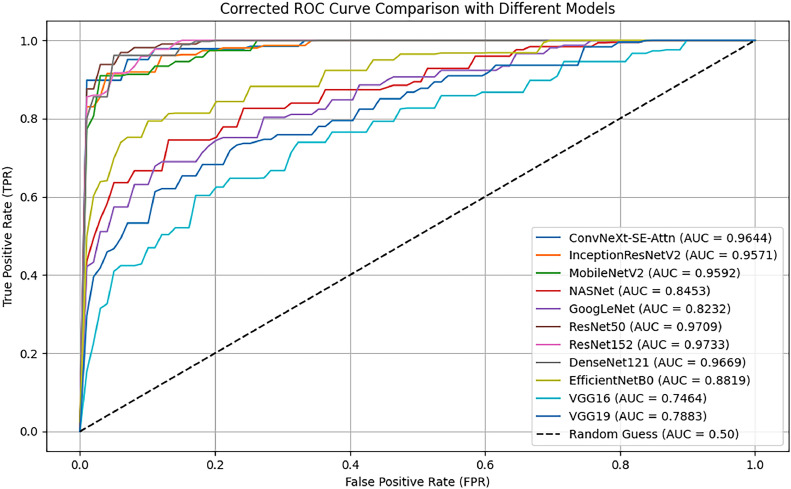
Receiver Operating Characteristic (ROC) curve comparison of different models, highlighting the superior AUC performance of ConvNeXt-SE-Attn.

The AUC-ROC curve (Fig. 10) compares the performance of multiple deep learning models. The proposed ConvNeXt-SE-Attn model achieves 0.9644 AUC score, showcasing its superior ability to distinguish between the three classes. Other models, including ResNet152, ResNet50, and DenseNet121, also exhibit stronger performance with 0.9733, 0.9709, 0.9669 AUC scores respectively. The VGG16 and VGG19 model, in contrast, has a significantly lower AUC, highlighting its weaker discriminatory power. The nearly perfect separation in ConvNeXt-SE-Attn’s ROC curve reaffirms its robustness in classifying oral cell types with high precision.

**Fig. 11:**
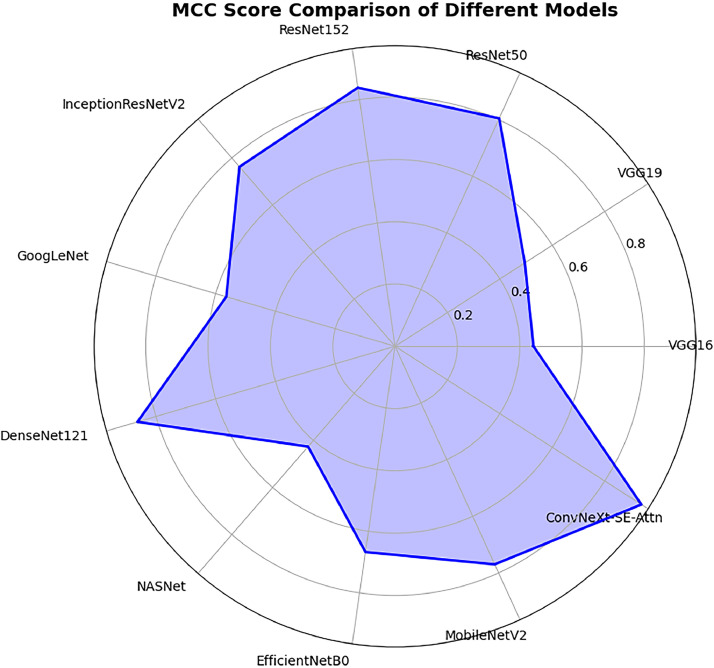
Matthews correlation coefficient (MCC) score comparison of different models.

Table 7 compares the performance of different deep learning models using two metrics: the MCC Score and the AUC Score. The MCC Score (Fig. 11) shows the overall quality of the model's predictions, while the AUC Score measures its ability to distinguish between classes. Higher values in both metrics indicate better performance. The table includes popular models like VGG16, ResNet50, DenseNet121, and proposed ConvNeXt-SE-Attn, making it easy to see which models perform best overall. Our proposed ConvNeXt-SE-Attn model achieved the highest MCC score of 0.9397 among all models.

**Table 7:** Comparison between proposed model with other deep learning models using MCC and AUC scores.

Model	MCC Score	AUC Score
VGG16	0.4443	0.7464
VGG19	0.4951	0.7883
ResNet50	0.8047	0.9709
ResNet152	0.8390	0.9733
InceptionResNetV2	0.7625	0.9571
GoogLeNet	0.5646	0.8232
DenseNet121	0.8616	0.9669
NASNet	0.4262	0.8453
EfficientNetB0	0.6674	0.8819
MobileNetV2	0.7697	0.9592
ConvNeXt-SE-Attn (Proposed)	0.9397	0.9644

The curves (Fig. 9) reveal that the ConvNeXt-SE-Attn model achieves near-ideal separation (0.9644), indicating exceptional class discriminative ability. In contrast, models like VGG16 (0.7464) and VGG19 (0.7883) show less distinctive curves, reflecting their relatively weaker performance in distinguishing subtle variations. Meanwhile, the models like EfficientNetB0 (0.8819), NASNet (0.8453), and GoogLeNet (0.8232) achieve moderate scores, and the advanced architectures like InceptionResNetV2 (0.9571), MobileNetV2 (0.9592), ResNet152 (0.9733), ResNet50 (0.9709), and DenseNet121 (0.9669) achieve higher scores.

The graph (Fig. 10) demonstrates that the ConvNeXt-SE-Attn model attains the highest MCC value (0.9397), underscoring its superior accuracy and reliability. In contrast, models like VGG16 (0.4443), VGG19 (0.4951), and NASNet (0.4262) show less reliability. This visualization further emphasizes the advantage of advanced architectures in consistently delivering robust performance in complex classification tasks like, ResNet152 (0.8390), ResNet50 (0.8047), and DenseNet121 (0. 8616).

### **Ablation study on** ConvNeXt-SE-Attn **model enhancements**

**Fig. 12:**
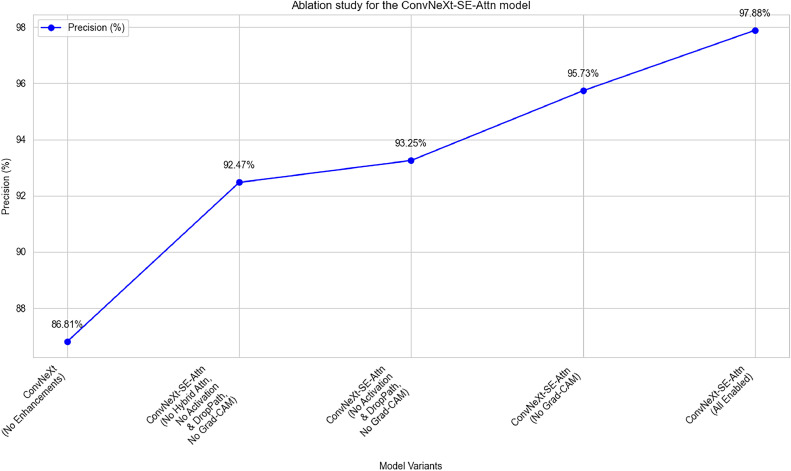
Ablation study for the proposed ConvNeXt-SE-Attn model.

An ablation experiment performed, that measured the independent value of each addition in ConvNeXt-SE-Attn model. The experimental method analyzed the model precision changes by sequentially disabling the SE blocks, hybrid attention mechanisms, advanced activations, optimizers and Grad-CAM one at a time. The analysis in Table 8, reveals that each enhancement in the ConvNeXt-SE-Attn model generated more precise results (Fig. 12), leading to the maximum precision with the complete configuration.

**Table 8:** Impact of ConvNeXt-SE-Attn model enhancements on precision across different approaches.

Model	Squeeze-and-Excitation	Hybrid Attention Mechanisms	GReLU + Swish + DropPath	Grad-CAM	Precision
ConvNeXt-SE-Attn	√	√	√	√	97.88 %
	√	√	√	×	95.73 %
	√	√	×	×	93.25 %
	√	×	×	×	92.47 %
	×	×	×	×	86.81 %

The proposed model's performance improved progressively with each enhancement. Without any enhancements, the baseline ConvNeXt backbone achieved a precision of 86.81 %. With the addition of Squeeze-and-Excitation (SE) blocks, precision increased to 92.47 %, and integrating Hybrid Attention Mechanisms further raised it to 93.25 %. Incorporating advanced activations such as GReLU and Swish along with a DropPath layer boosted precision to 95.73 %. Finally, the use of Grad-CAM in the classification process resulted in a final precision of 97.88 %. Overall, these incremental improvements contributed to an 11.07 % increase in precision from the baseline model to the final ConvNeXt-SE-Attn model.

### Limitations

The performance of the proposed model is better than compare to the benchmark deep learning models. However, some of the limitations are identified and mentioned below.

• The model training process becomes longer and consumes more resources because the combination of SE blocks, hybrid attention mechanisms, and advanced activation functions creates computational complexity that increases training times.
• Complex architecture within the model supports precision but causes decreased model interpretability which requires extra testing and validation as a prerequisite for real-world medical implementation.
• The specialized design for oral cell types, demonstrated potential limitations when trying to generalize to wider or different cancer imaging applications.
• While the in vitro results are promising, the generalisability of the proposed model to real-world clinical scenarios, such as biopsy-based diagnostics, remains untested and requires future validation.
• The model's many advanced parts make it hard to pinpoint and fix specific errors, which can complicate efforts to improve and optimize it over time.

## Ethics statements

The research did not involve human participants or animal subjects, hence, it did not require approval from an institutional or national research ethics committee.

## Declaration of competing interest

The authors declare that they have no known competing financial interests or personal relationships that could have appeared to influence the work reported in this paper.

## Data Availability

The research data used in this study mentioned in the paper with link.
